# Supplementary material for: Machine learning-guided realization of full-color high-quantum-yield carbon quantum dots
Source: Nat Commun. 2024 Jun 6;15:4843. doi: 10.1038/s41467-024-49172-6 (PMC11156924; doi:10.1038/s41467-024-49172-6)
Supplement: Supplementary file 1 — Supplementary Information [file 41467_2024_49172_MOESM1_ESM.pdf]

Supplementary Information for:

Machine learning-guided realization of full-color high-quantum-yield carbon quantum dots

Huazhang Guo[1], Yuhao Lu[2], Zhendong Lei[3], Hong Bao[1], Mingwan Zhang[1], Zeming Wang[1], Cuntai Guan[2✉], Bijun Tang[3✉], Zheng Liu[3,4,5✉] & Liang Wang[1,3✉]

[1]Institute of Nanochemistry and Nanobiology, School of Environmental and Chemical Engineering, Shanghai University, 99 Shangda Road, BaoShan District, Shanghai 200444, P. R. China

[2]College of Computing and Data Science, Nanyang Technological University, 50 Nanyang Avenue, Singapore 639798, Singapore

[3]School of Materials Science and Engineering, Nanyang Technological University, 50 Nanyang Avenue, Singapore 639798, Singapore

[4]CINTRA CNRS/NTU/THALES, UMI 3288, Research Techno Plaza, 50 Nanyang Drive, Border X Block, Level 6, Singapore 637553, Singapore

[5]Institute for Functional Intelligent Materials, National University of Singapore, Singapore, Singapore

These authors contributed equally: Huazhang Guo, Yuhao Lu, Zhendong Lei, Hong Bao

✉E-mail: ctguan@ntu.edu.sg; bjtang@ntu.edu.sg; z.Liu@ntu.edu.sg; wangl@shu.edu.cn

Table of Contents

Supplementary Figures

Supplementary Fig. 1 | Flowchart of ML-guided synthesis of panchromatic CQDs.

Supplementary Fig. 2 | Schematic diagram of the parameter regulation process for synthesizing CQDs.

Supplementary Fig. 3 | Nested cross validation results of four candidate ML models.

Supplementary Fig. 4 | Histogram of critical points of 100 times repeated offline XGBoost-guided hydrothermal synthesis.

Supplementary Fig. 5 | Target properties (PL wavelength and PLQY) versus the iterations.

Supplementary Fig. 6 | Optical properties of full-color fluorescent CQDs.

Supplementary Fig. 7 | Three-dimensional fluorescence properties of full-color fluorescent CQDs.

Supplementary Fig. 8 | Optical characterizations of full-color fluorescent CQDs.

Supplementary Fig. 9 | Storage stability characterizations of full-color fluorescent CQDs with different times.

Supplementary Fig. 10 | Ultraviolet photoelectron spectroscopy spectra of full-color fluorescent CQDs.

Supplementary Fig. 11 | XRD spectra of full-color fluorescent CQDs.

Supplementary Fig. 12 | Raman spectra of full-color fluorescent CQDs.

Supplementary Fig. 13 | FT-IR spectra of full-color fluorescent CQDs.

Supplementary Fig. 14 | XPS characterizations of p-CQDs.

Supplementary Fig. 15 | XPS characterizations of b-CQDs.

Supplementary Fig. 16 | XPS characterizations of c-CQDs.

Supplementary Fig. 17 | XPS characterizations of dc-CQDs.

Supplementary Fig. 18 | XPS characterizations of g-CQDs.

Supplementary Fig. 19 | XPS characterizations of yg-CQDs.

Supplementary Fig. 20 | XPS characterizations of y-CQDs.

Supplementary Fig. 21 | XPS characterizations of o-CQDs.

Supplementary Fig. 22 | XPS characterizations of or-CQDs.

Supplementary Fig. 23 | XPS characterizations of r-CQDs.

Supplementary Fig. 24 | TEM characterizations of full-color fluorescent CQDs.

Supplementary Tables

Supplementary Table 1 | Value ranges of input synthesis parameters.

Supplementary Table 2 | Value ranges of colors defined by PL wavelength.

Supplementary Table 3 | PL scan conditions and PLQY data for full-color fluorescent CQDs.

Supplementary Table 4 | Recent progress of PL wavelengths of red CQDs.

Supplementary Table 5 | The energy levels of full-color fluorescent CQDs.

Supplementary References

Supplementary Figures

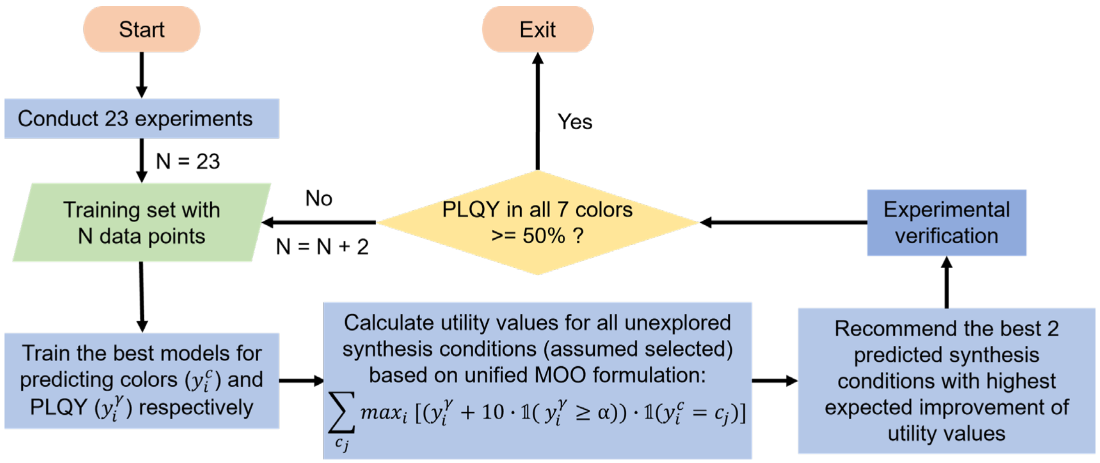

Supplementary Fig. 1 | Flowchart of machine learning (ML)-guided synthesis of panchromatic carbon quantum dots (CQDs). Initially, a training set is collected with random selected synthesis conditions, and each condition is labelled by photoluminescence (PL) wavelength/ PL quantum yields (PLQY) from manual experimentation and characterization. Color labels (y_i^c) are given from discretizing predicted PL wavelength as shown in Supplementary Table 2. The size of initial training set (i.e., $N = 23$) is jointly determined by two requirements: 1) the minimum number of data points (i.e., 20) required to run cross validation of ML surrogate models for hyperparameter searching, 2) the need to verify the viability of synthesizing full-color CQDs with this synthesis search space. Upon the 23rd randomly selected synthesis condition, synthesized CQDs exhibited versatile fluorescent colors with longspan emission wavelength data, achieving both requirements. After collecting the initial training set, two ML surrogate models with best hyperparameters are trained for predicting PL wavelength (or colors) and PLQY respectively. The two trained models are then employed to predict on all unexplored synthesis conditions. Then the corresponding utility values of these unexplored conditions are computed based on multi-objective optimization (MOO) objective function, through assuming that this one condition will be selected and augmented to the existing dataset of size N. Out of all unexplored conditions, the two with highest expected improvement of utility values are recommended for experimental verification and characterization. If the maximum PLQYs in all seven colors are larger or equal to 50%, the iterative ML-driven MOO loops end, otherwise the iteration proceeds and the two newly

synthesized data points are augmented into the training set (i.e., $N = N + 2$) for the next round of model training and synthesis condition recommendation.

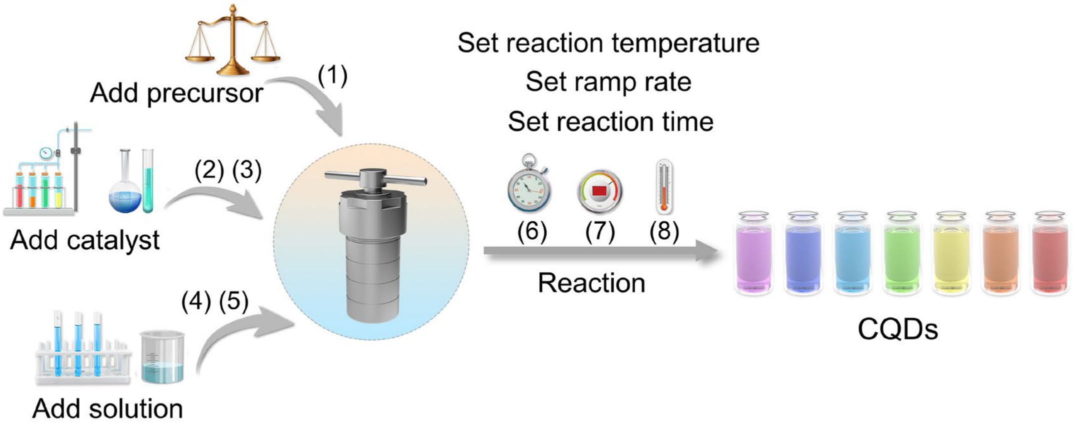

Supplementary Fig. 2 | Schematic diagram of the parameter regulation process for synthesizing CQDs. There are eight primary control parameters in the synthesis process, including: (1) mass of precursor, (2) type of catalyst, (3) volume/mass of catalyst, (4) type of solution, (5) volume of solution, (6) reaction temperature, (7) ramp rate and (8) reaction time. These control parameters are involved in different steps of the synthesis process depicted. The detailed value ranges of the synthesis parameters are listed in Supplementary Table 1.

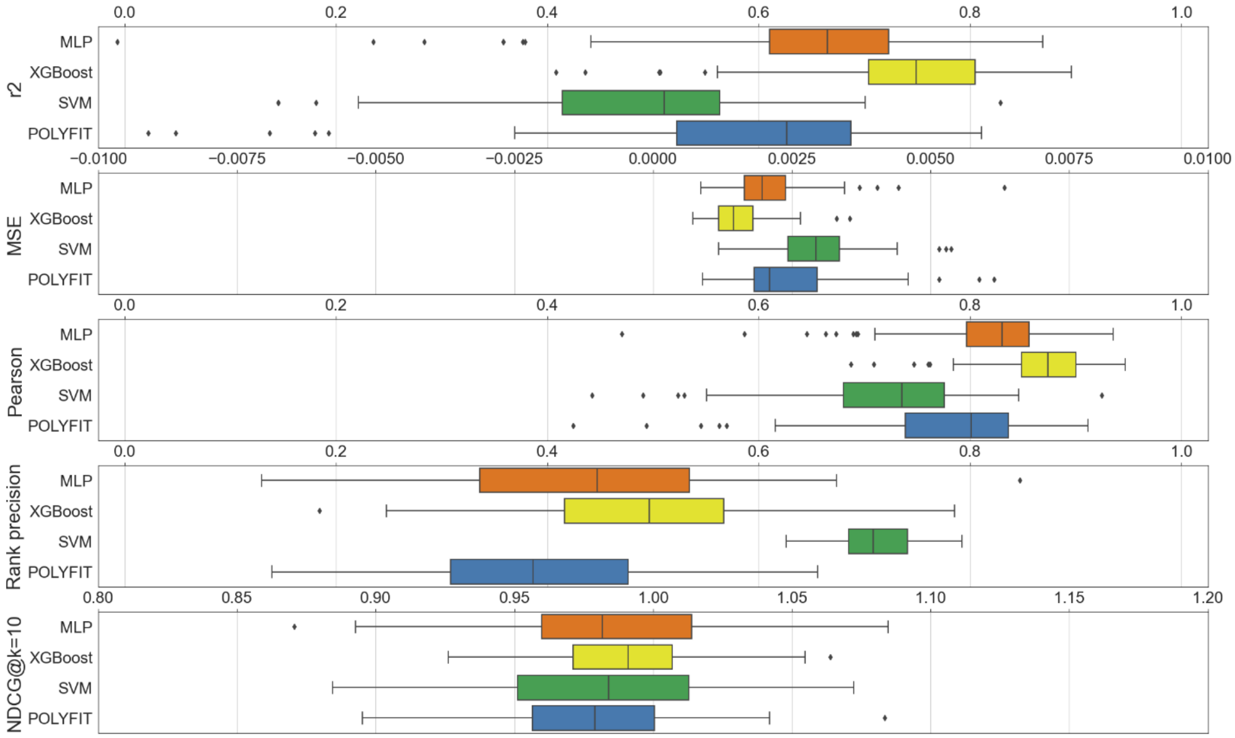

Supplementary Fig. 3 | Nested cross validation results of four candidate ML models. The surrogate ML model selection results of nested cross validation on the hydrothermal synthesis dataset extracted from our previous study[1]. As shown above, XGBoost outperforms MLP, SVM and POLYFIT[2-6] in terms of mean coefficient of determination (r^2), MSE, Pearson and normalized discounted cumulative gain (NDCG). Therefore, XGBoost is chosen as the surrogate ML model to predict the target properties for this work. The box ranges from 25th percentile (Q1) to 75th percentile (Q3), with the middle line marking the median. The whiskers represent the lowest and highest values, and the diamonds indicate the outliers. The outliers are identified as values above Q3+1.5× IQR, or below Q1-1.5× IQR, whereas IQR = Q3 – Q1.

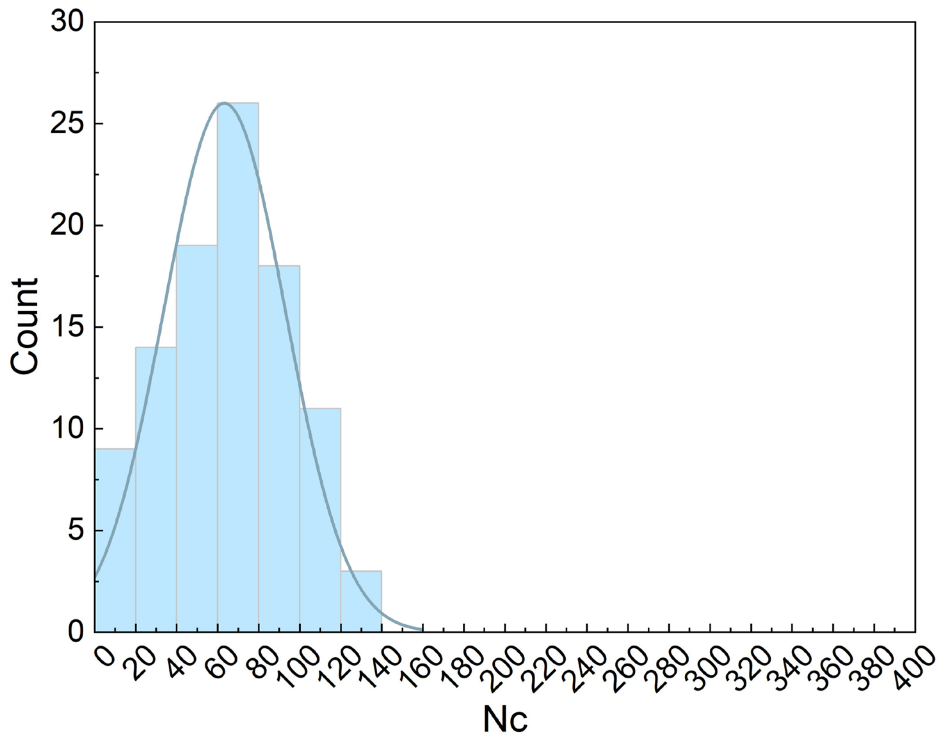

Supplementary Fig. 4 | Histogram of critical points of 100 times repeated offline XGBoost-guided hydrothermal synthesis. For each offline trial, it is assumed that the search space is constrained in the 391 data samples collected in our previous work of ML-guided hydrothermal synthesis for enhancing PLQY only[1]. At the start of each trial, 20 data points are randomly sampled as the initial training set excluding the one with maximum PLQY (i.e., optimal condition) of the whole dataset, while the other 371 (391-20=371) synthesis conditions are deemed unexplored. Then, a simulated process of XGBoost-guided synthesis process begins. XGBoost model is trained on the given training set, and then recommends the top 2 candidates based on the predicted PLQY of the unexplored conditions. The selected 2 data samples are labelled with the real PLQY and augmented to the training set for the next iteration. The recommendation loops end at where the optimal condition is found, and Nc the number of conditions newly explored under the guidance of XGBoost model in this trial. The offline trial is repeated 100 times and the distribution of these 100 Nc is illustrated in the histogram.

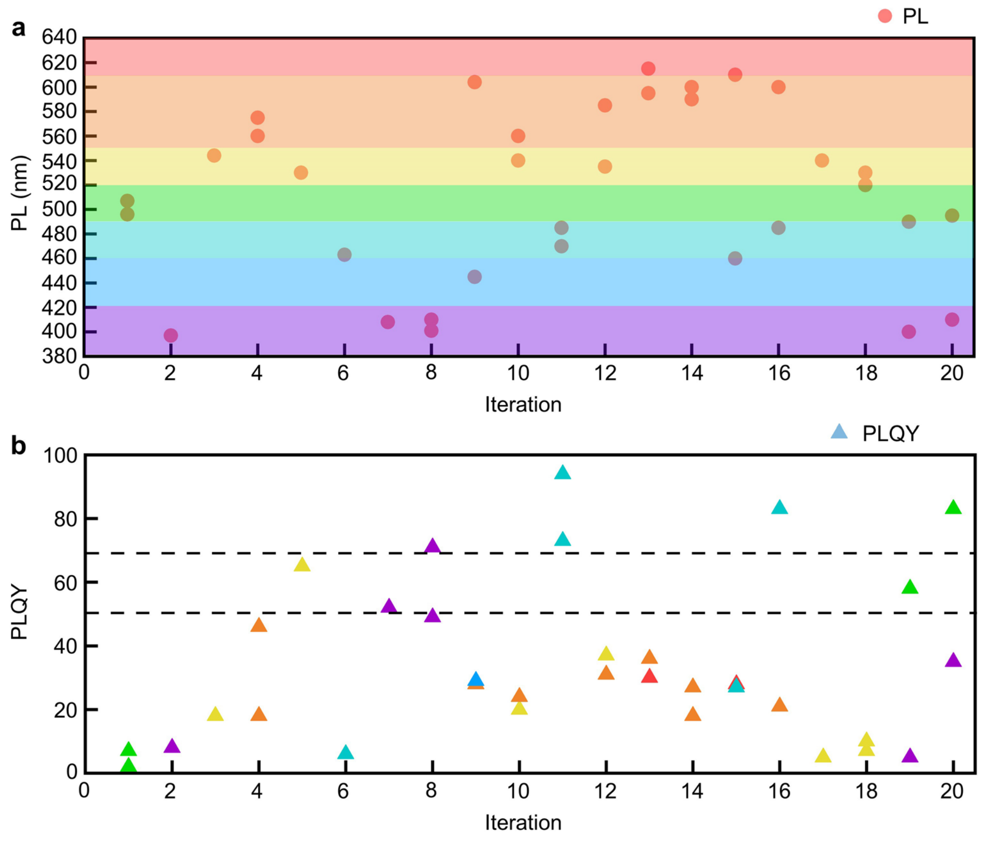

Supplementary Fig. 5 | Target properties (PL wavelength and PLQY) versus the iterations. PL wavelength (**a**) and PLQY (**b**) of CQDs are plotted versus the iterations. The colors of triangles in (b) reflect the color of the CQDs samples, as defined in Supplementary Table 2.

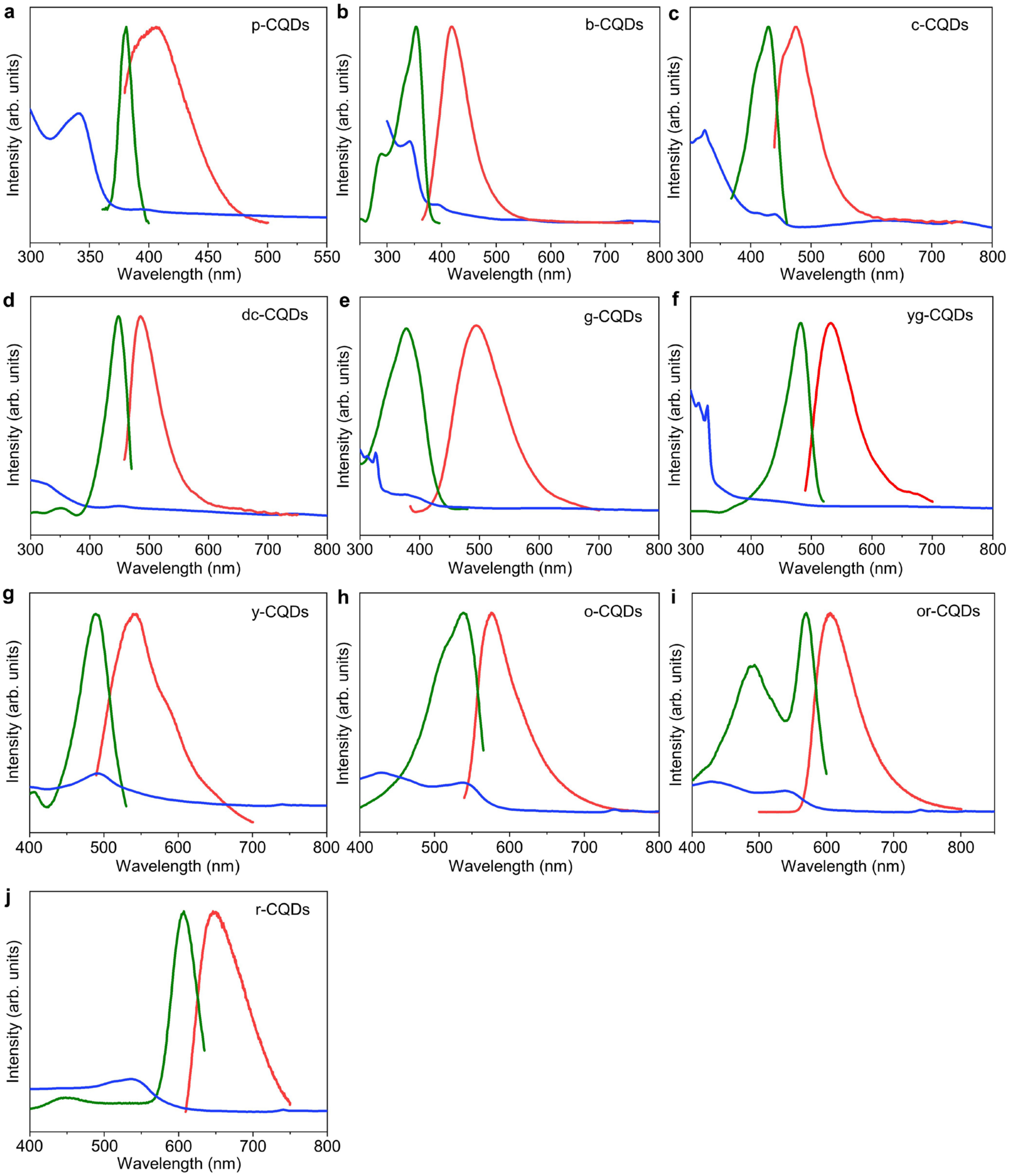

Supplementary Fig. 6 | Optical properties of full-color fluorescent CQDs. UV-Vis absorption (blue), PL (red) and PLE (green) spectra of p-CQDs (**a**), b-CQDs (**b**), c-CQDs (**c**), dc-CQDs (**d**), g-CQDs (**e**), yg-CQDs (**f**), y-CQDs (**g**), o-CQDs (**h**), or-CQDs (**i**) and r-CQDs (**j**), respectively.

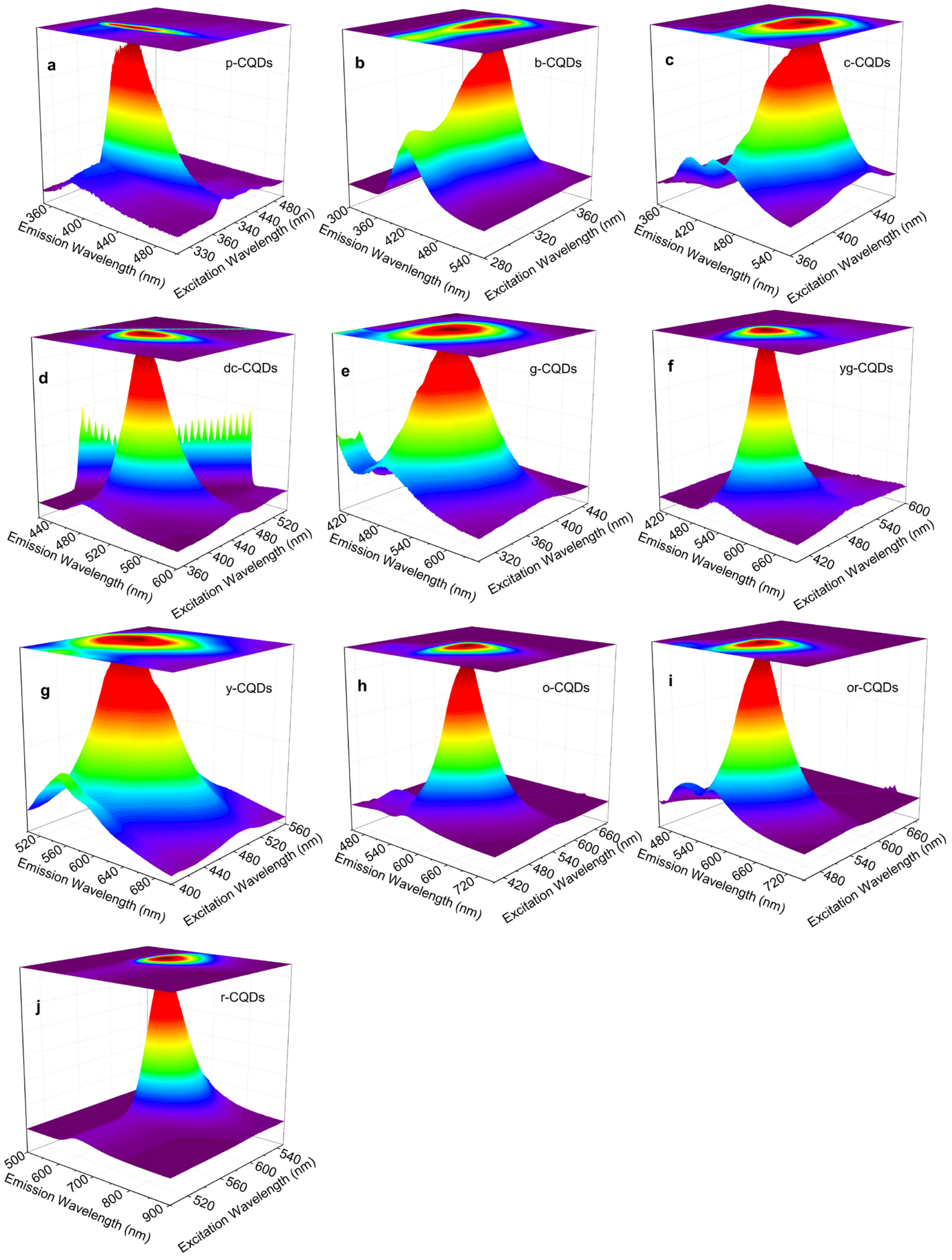

Supplementary Fig. 7 | Three-dimensional fluorescence properties of full-color fluorescent CQDs. Three-dimensional fluorescence spectra of p-CQDs (**a**), b-CQDs (**b**), c-CQDs (**c**), dc-CQDs (**d**), g-CQDs (**e**), yg-CQDs (**f**), y-CQDs (**g**), o-CQDs (**h**), or-CQDs (**i**) and r-CQDs (**j**), respectively.

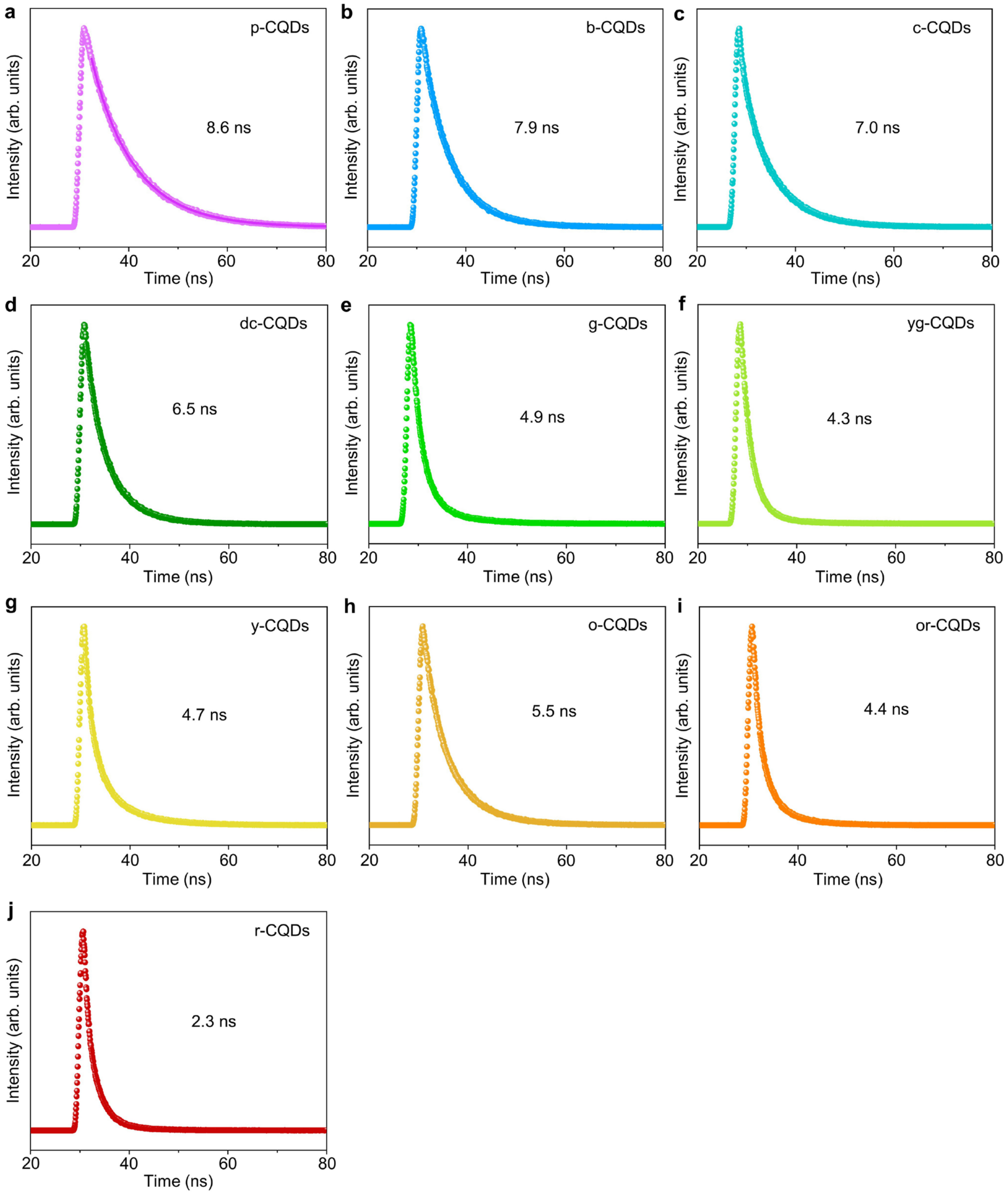

Supplementary Fig. 8 | Optical characterizations of full-color fluorescent CQDs. Time-resolved PL spectra of p-CQDs (**a**), b-CQDs (**b**), c-CQDs (**c**), dc-CQDs (**d**), g-CQDs (**e**), yg-CQDs (**f**), y-CQDs (**g**), o-CQDs (**h**), or-CQDs (**i**) and r-CQDs (**j**), respectively.

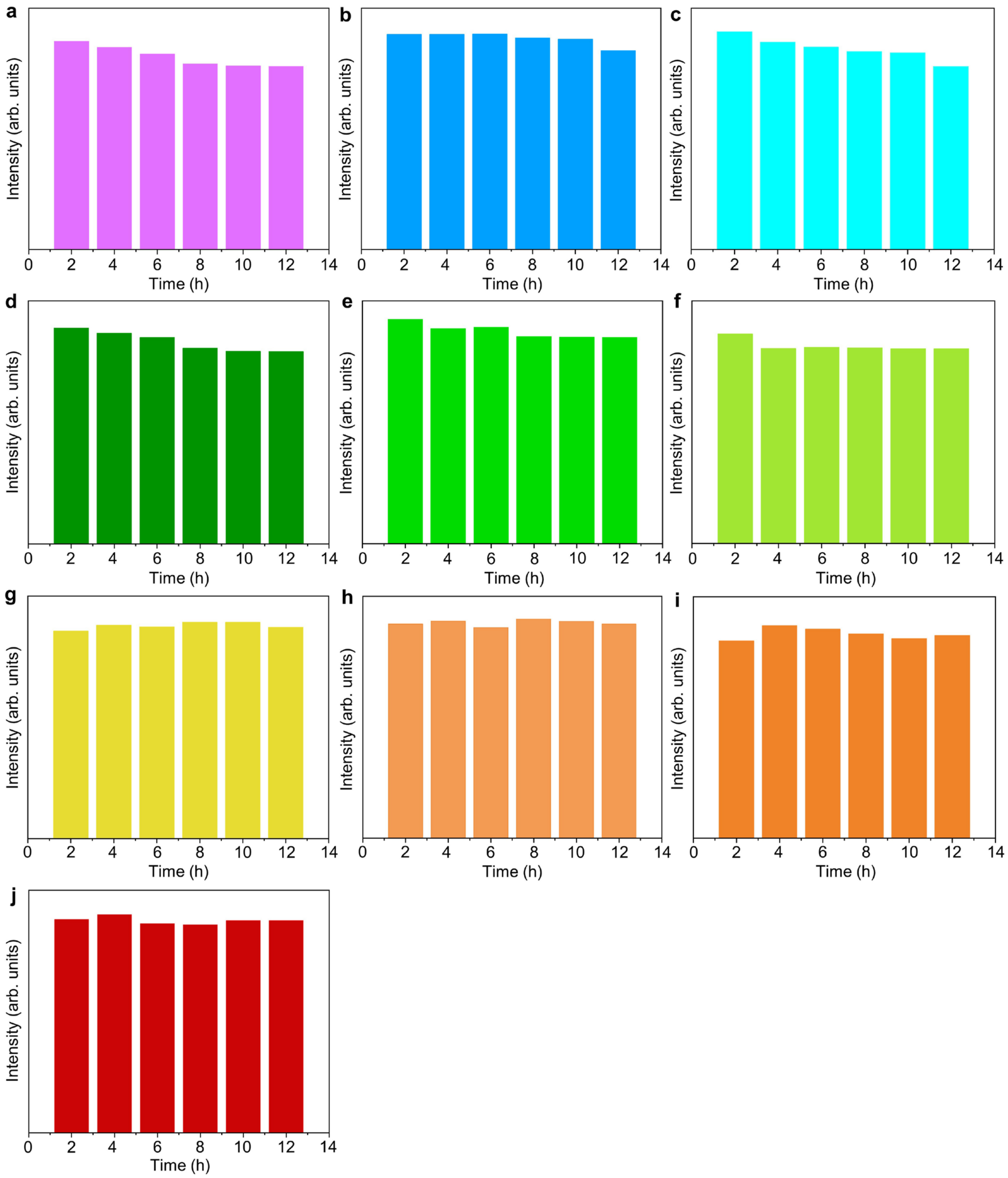

Supplementary Fig. 9 | Storage stability characterizations of full-color fluorescent CQDs with different times. Storage stability test of p-CQDs (**a**), b-CQDs (**b**), c-CQDs (**c**), dc-CQDs (**d**), g-CQDs (**e**), yg-CQDs (**f**), y-CQDs (**g**), o-CQDs (**h**), or-CQDs (**i**) and r-CQDs (**j**), respectively.

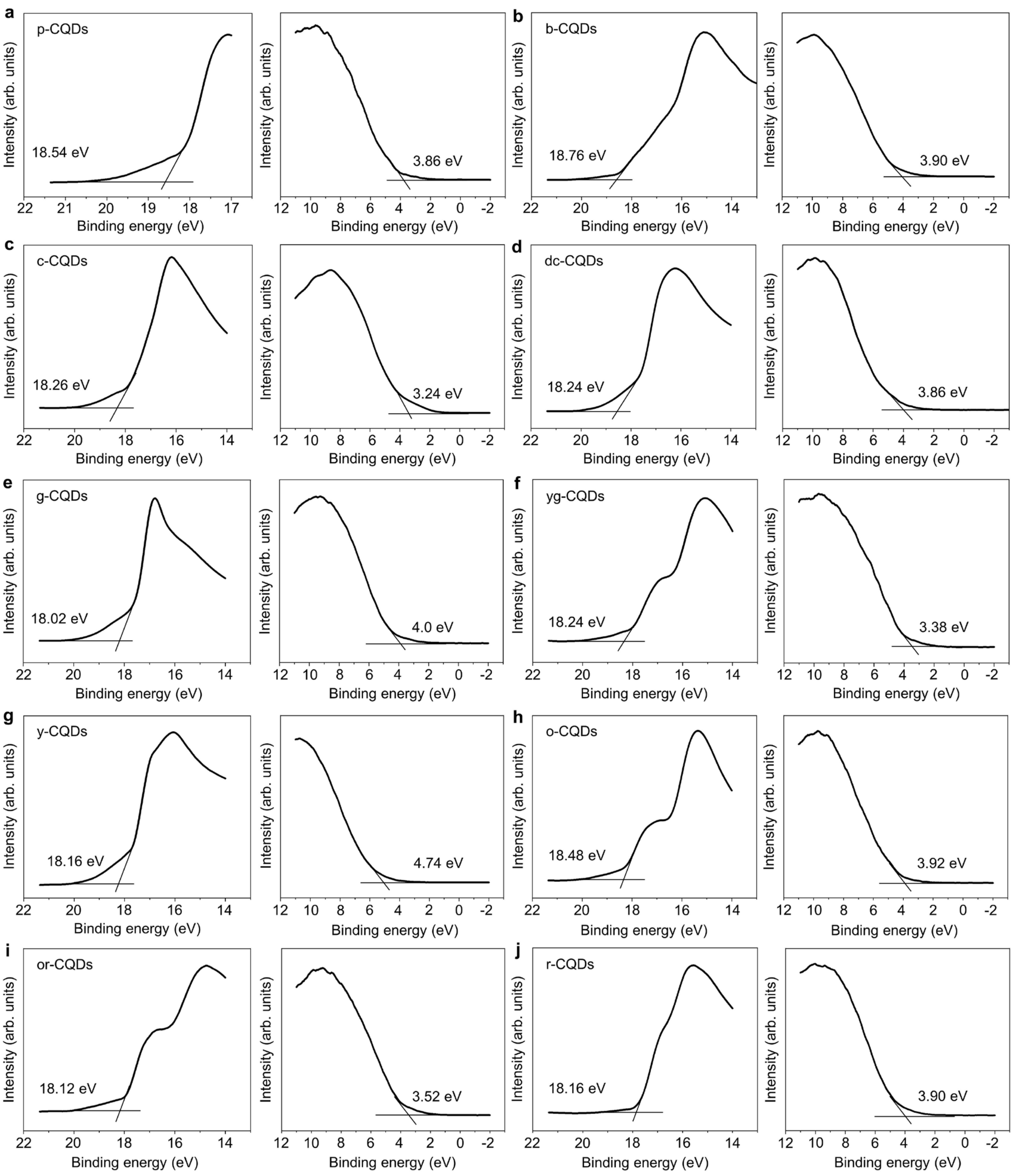

Supplementary Fig. 10 | Ultraviolet photoelectron spectroscopy spectra of full-color fluorescent CQDs. Ultraviolet photoelectron spectroscopy data of p-CQDs (**a**), b-CQDs (**b**), c-CQDs (**c**), dc-CQDs (**d**), g-CQDs (**e**), yg-CQDs (**f**), y-CQDs (**g**), o-CQDs (**h**), or-CQDs (**i**) and r-CQDs (**j**), respectively.

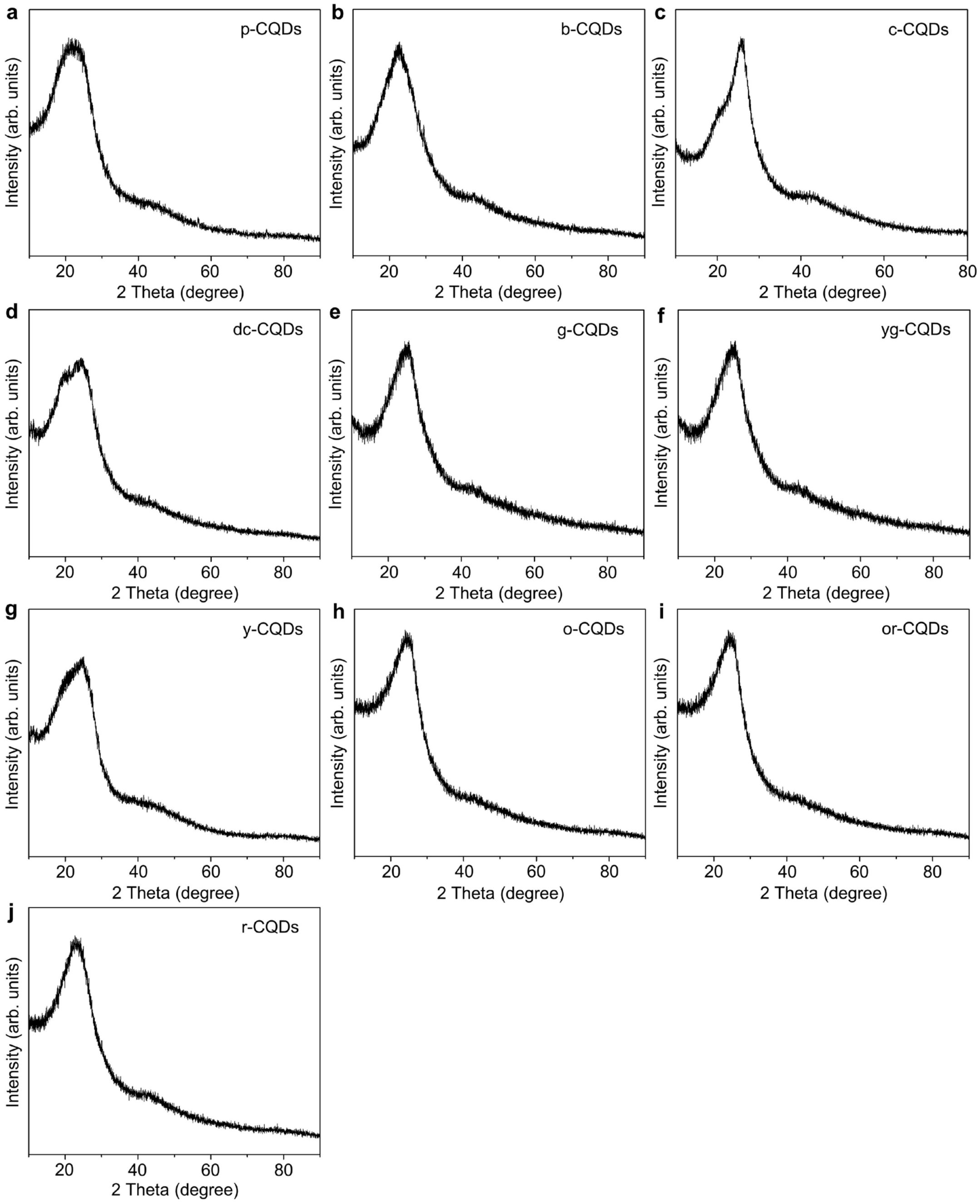

Supplementary Fig. 11 | XRD spectra of full-color fluorescent CQDs. XRD spectra of p-CQDs (**a**), b-CQDs (**b**), c-CQDs (**c**), dc-CQDs (**d**), g-CQDs (**e**), yg-CQDs (**f**), y-CQDs (**g**), o-CQDs (**h**), or-CQDs (**i**) and r-CQDs (**j**), respectively.

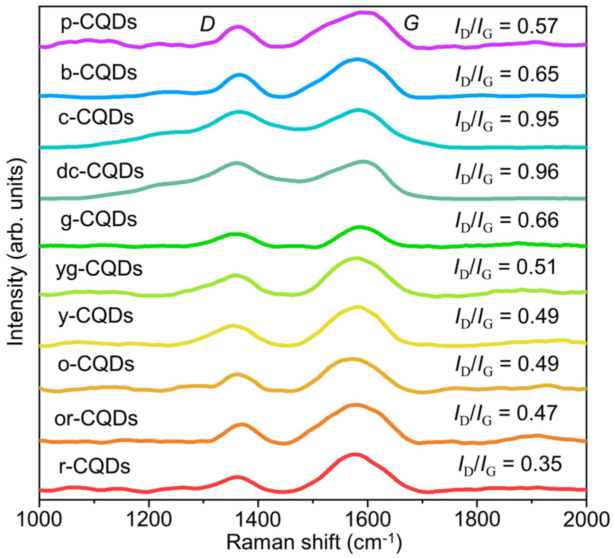

Supplementary Fig. 12 | Raman spectra of full-color fluorescent CQDs. Raman spectra of p-CQDs, b-CQDs, c-CQDs, dc-CQDs, g-CQDs, yg-CQDs, y-CQDs, o-CQDs, or-CQDs and r-CQDs, respectively.

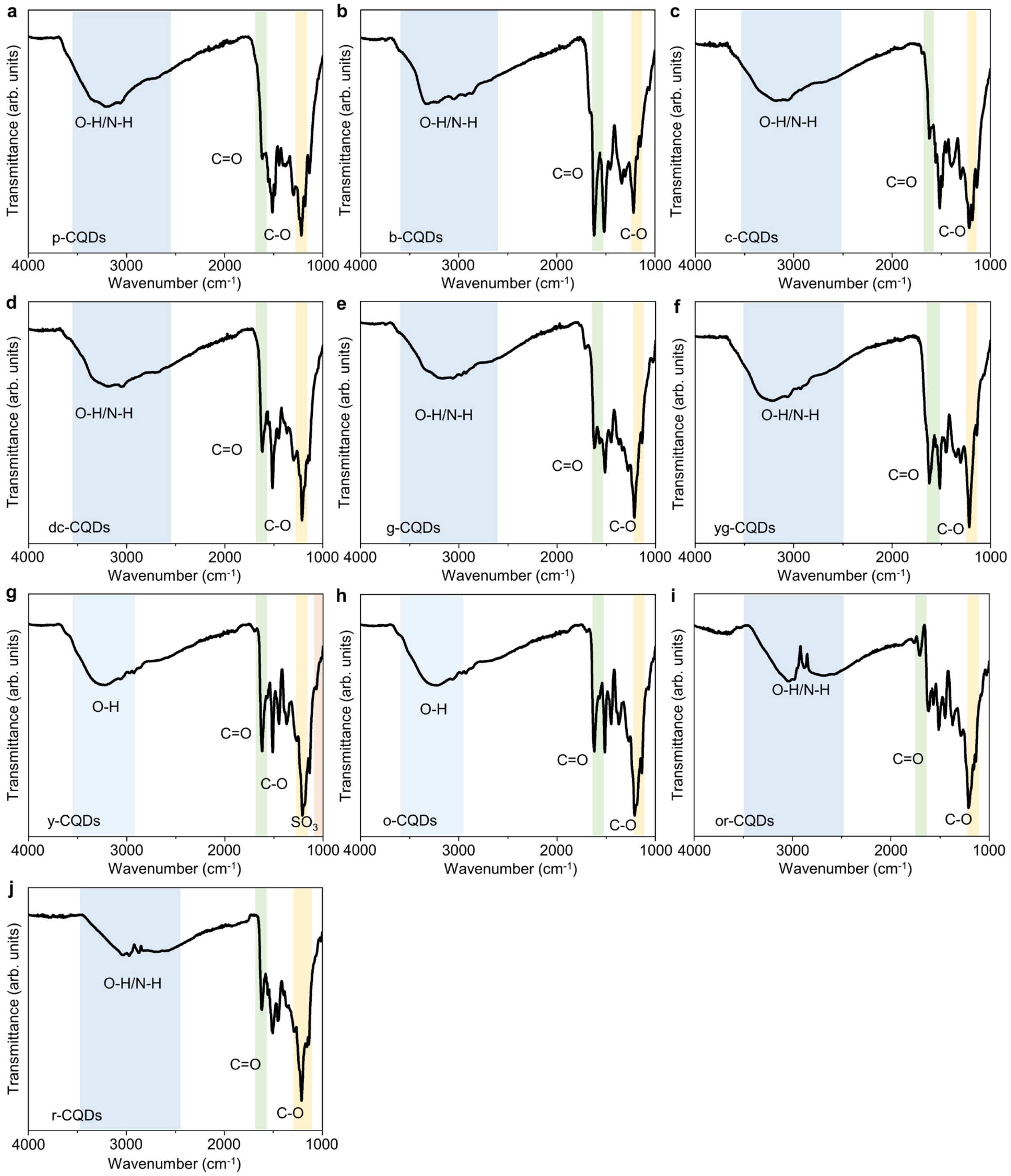

Supplementary Fig. 13 | FT-IR spectra of full-color fluorescent CQDs. FT-IR spectra of p-CQDs (**a**), b-CQDs (**b**), c-CQDs (**c**), dc-CQDs (**d**), g-CQDs (**e**), yg-CQDs (**f**), y-CQDs (**g**), o-CQDs (**h**), or-CQDs (**i**) and r-CQDs (**j**), respectively. The corresponding functional groups of CQDs are highlighted using shaded regions.

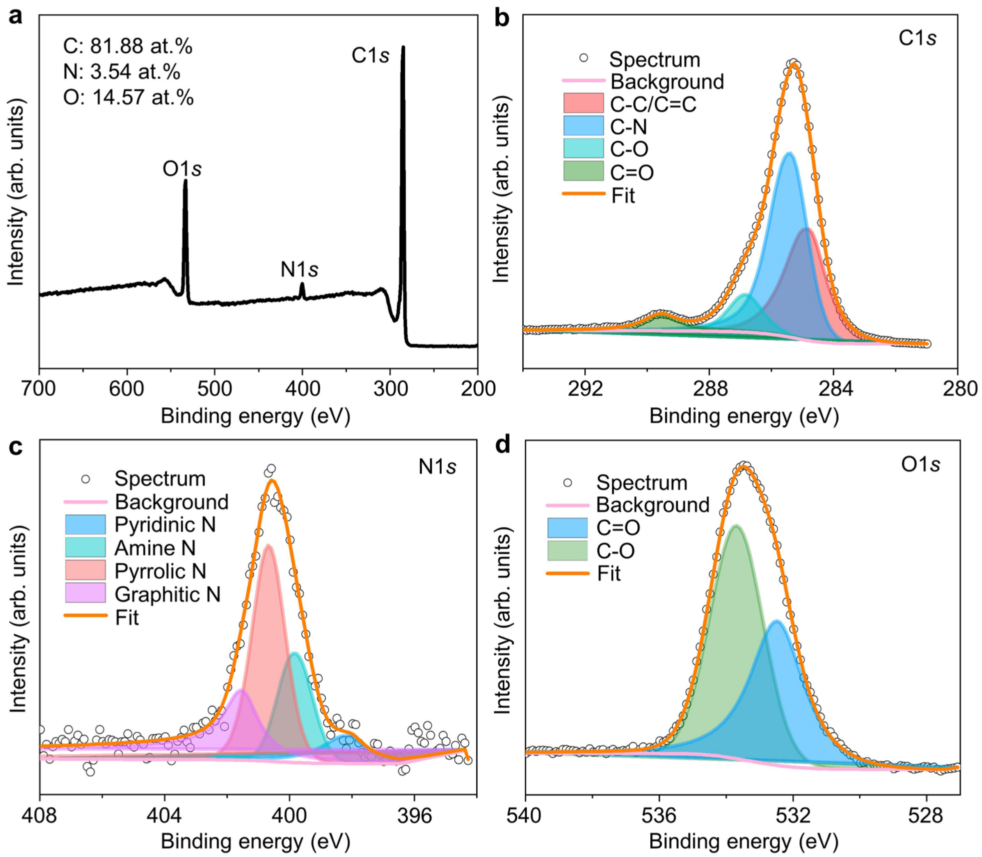

Supplementary Fig. 14 | XPS characterizations of p-CQDs. (**a**) XPS survey, (**b**) high-resolution C1*s*, (**c**) high-resolution N1*s*, (**d**) high-resolution O1*s* spectra of the p-CQDs.

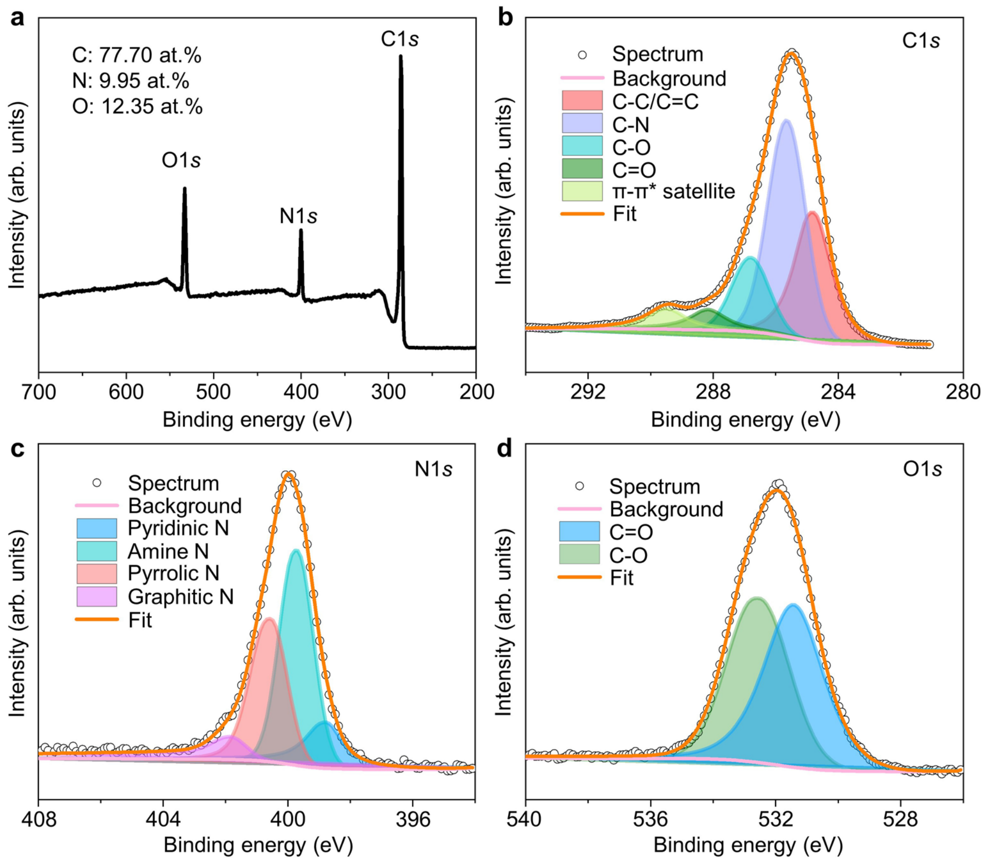

Supplementary Fig. 15 | XPS characterizations of b-CQDs. (**a**) XPS survey, (**b**) high-resolution C1*s*, (**c**) high-resolution N1*s*, (**d**) high-resolution O1*s* spectra of the b-CQDs.

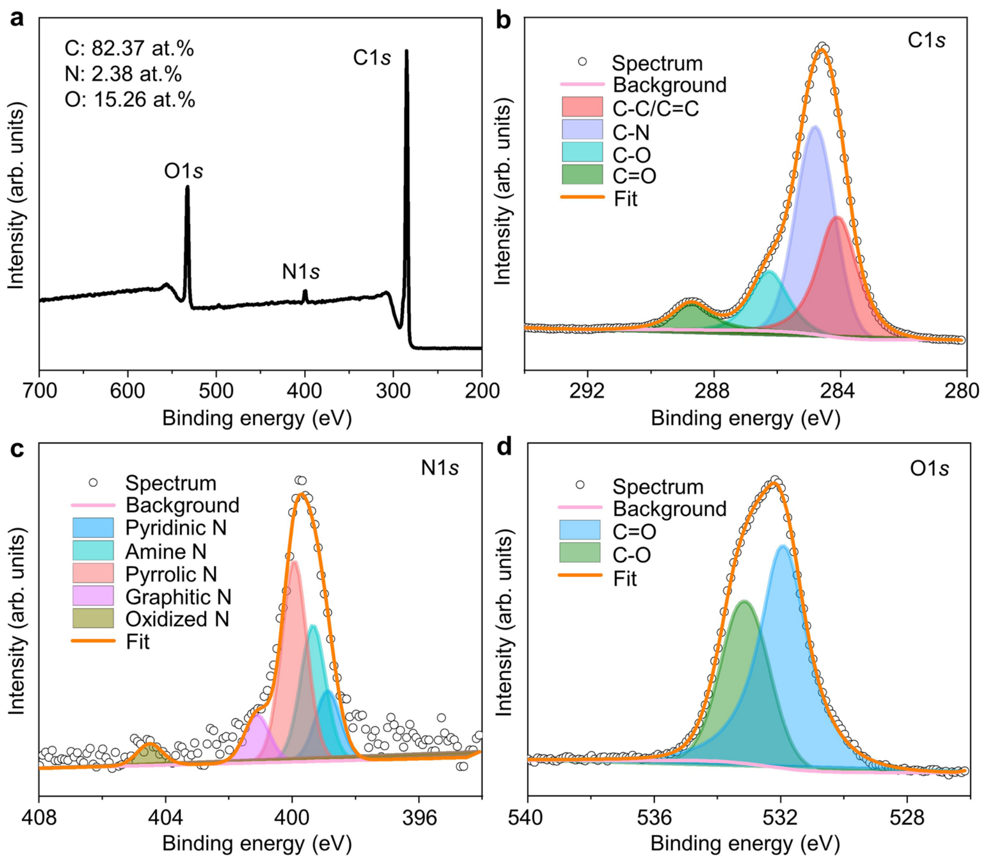

Supplementary Fig. 16 | XPS characterizations of c-CQDs. (**a**) XPS survey, (**b**) high-resolution C1*s*, (**c**) high-resolution N1*s*, (**d**) high-resolution O1*s* spectra of the c-CQDs.

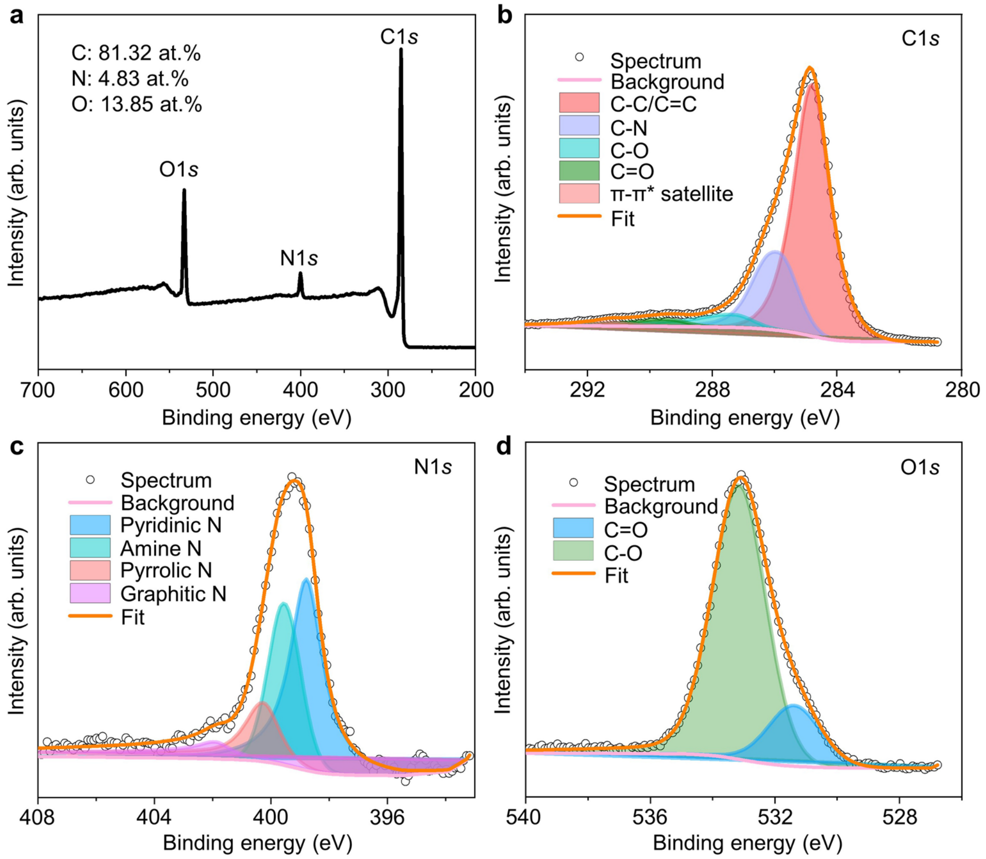

Supplementary Fig. 17 | XPS characterizations of dc-CQDs. (**a**) XPS survey, (**b**) high-resolution C1*s*, (**c**) high-resolution N1*s*, (**d**) high-resolution O1*s* spectra of the dc-CQDs.

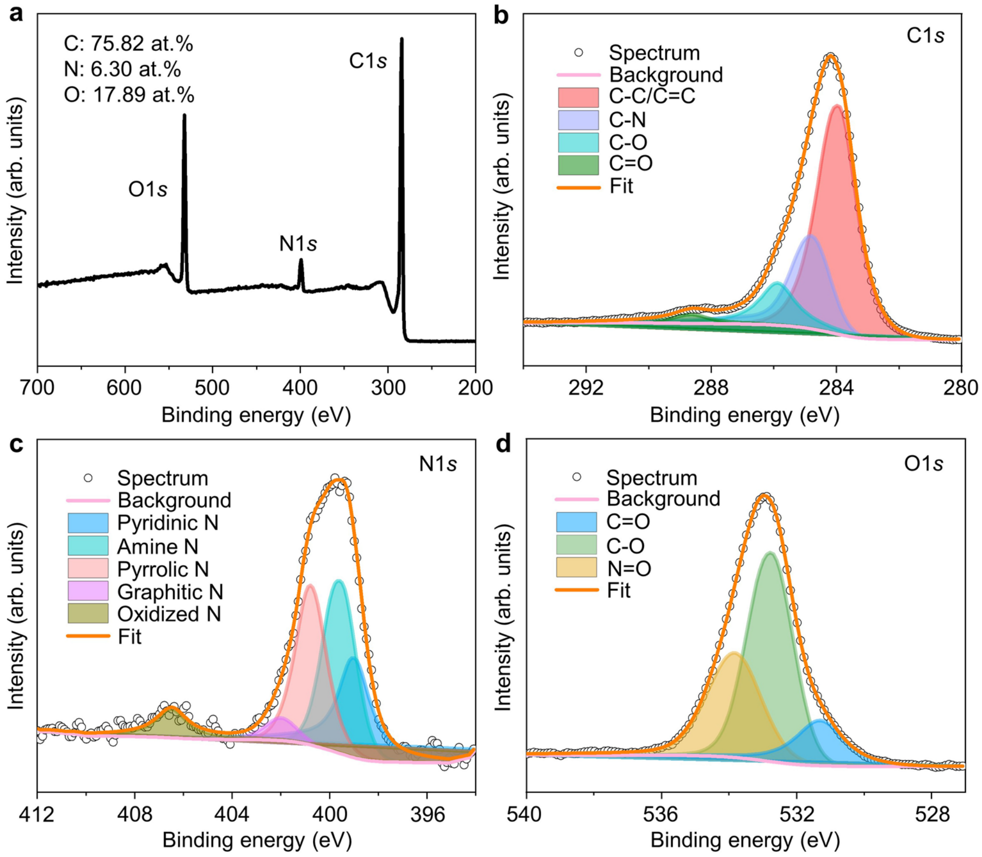

Supplementary Fig. 18 | XPS characterizations of g-CQDs. (**a**) XPS survey, (**b**) high-resolution C1*s*, (**c**) high-resolution N1*s*, (**d**) high-resolution O1*s* spectra of the g-CQDs.

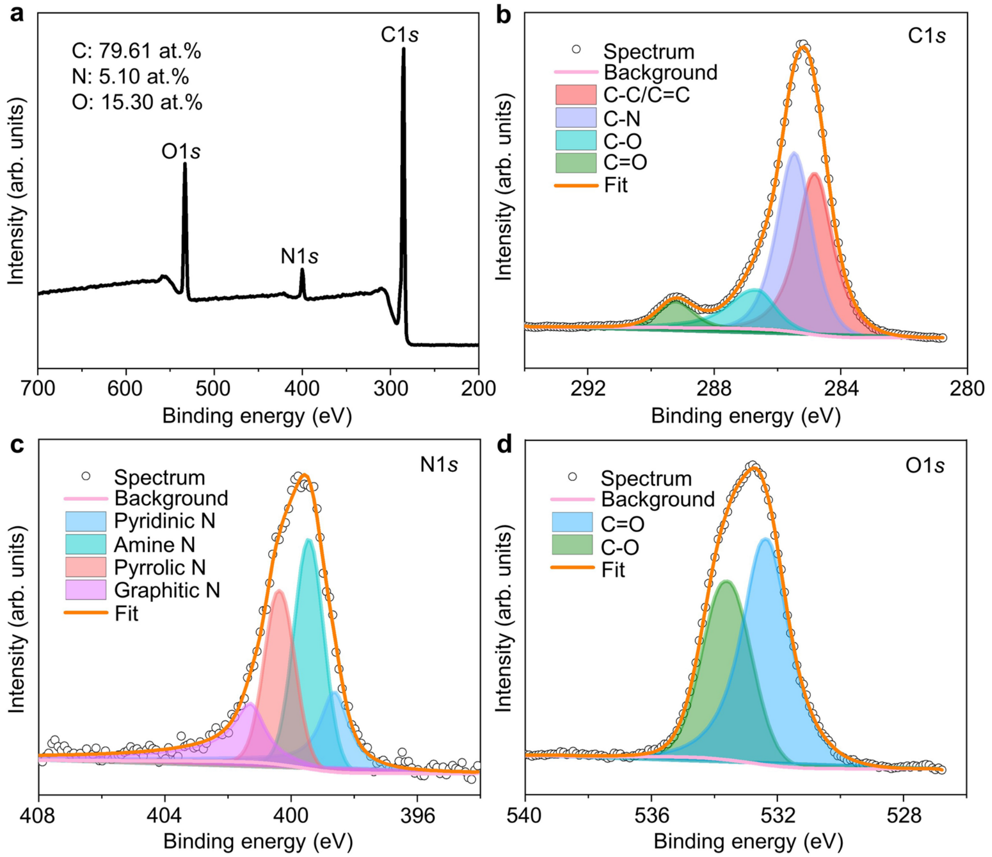

Supplementary Fig. 19 | XPS characterizations of yg-CQDs. (**a**) XPS survey, (**b**) high-resolution C1*s*, (**c**) high-resolution N1*s*, (**d**) high-resolution O1*s* spectra of the yg-CQDs.

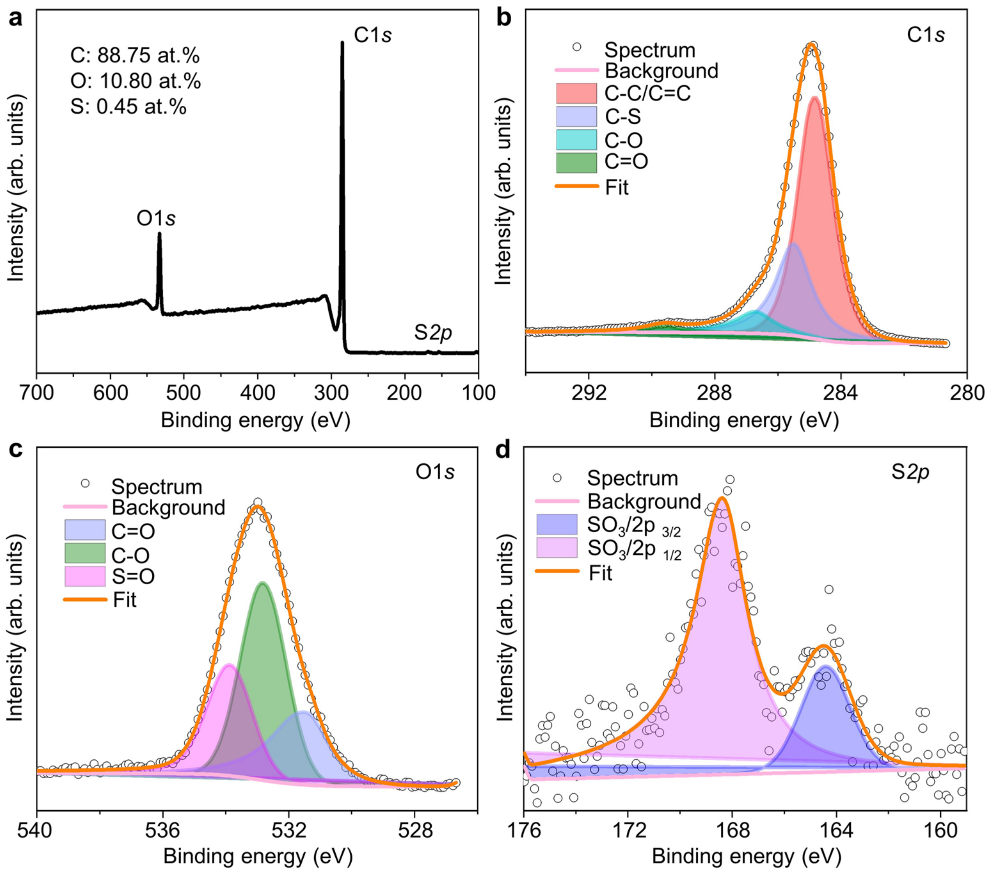

Supplementary Fig. 20 | XPS characterizations of y-CQDs. (**a**) XPS survey, (**b**) high-resolution C1*s*, (**c**) high-resolution O1*s*, (**d**) high-resolution S2*p* spectra of the y-CQDs.

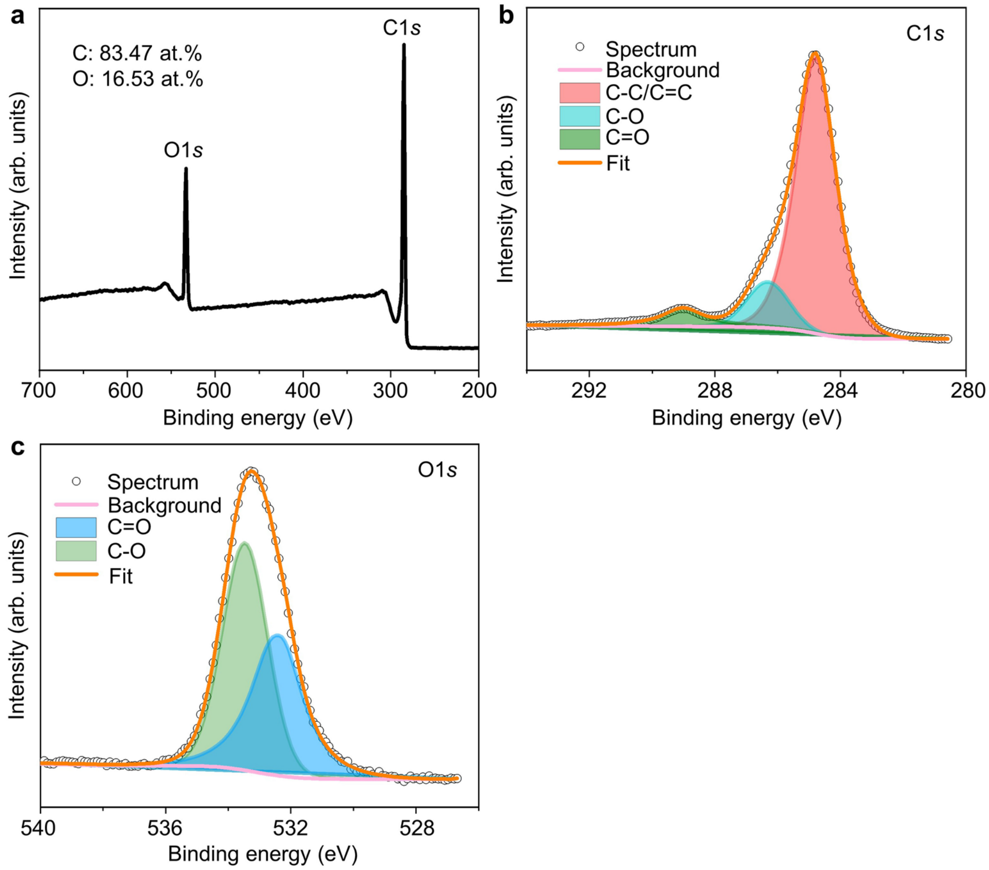

Supplementary Fig. 21 | XPS characterizations of o-CQDs. (**a**) XPS survey, (**b**) high-resolution C1*s*, (**c**) O1*s* spectra of the o-CQDs.

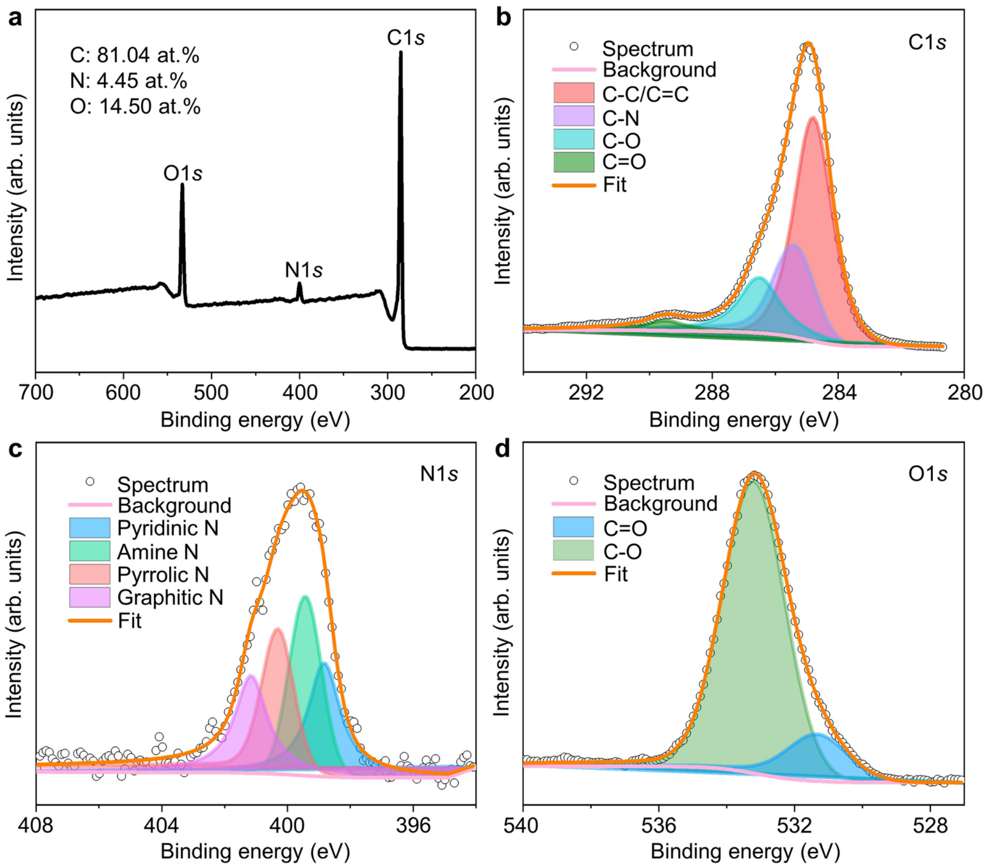

Supplementary Fig. 22 | XPS characterizations of or-CQDs. (**a**) XPS survey, (**b**) high-resolution C1*s*, (**c**) high-resolution N1*s*, (**d**) high-resolution O1*s* spectra of the or-CQDs.

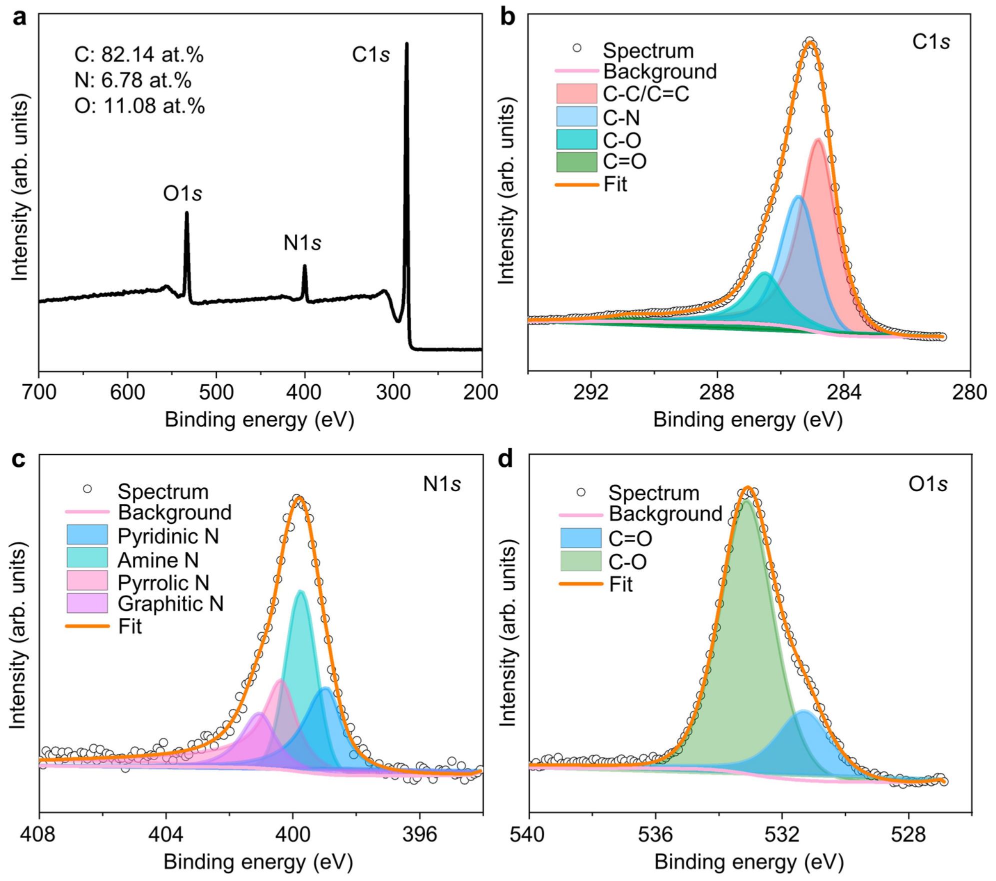

Supplementary Fig. 23 | XPS characterizations of r-CQDs. (**a**) XPS survey, (**b**) high-resolution C1*s*, (**c**) high-resolution N1*s*, (**d**) high-resolution O1*s* spectra of the r-CQDs.

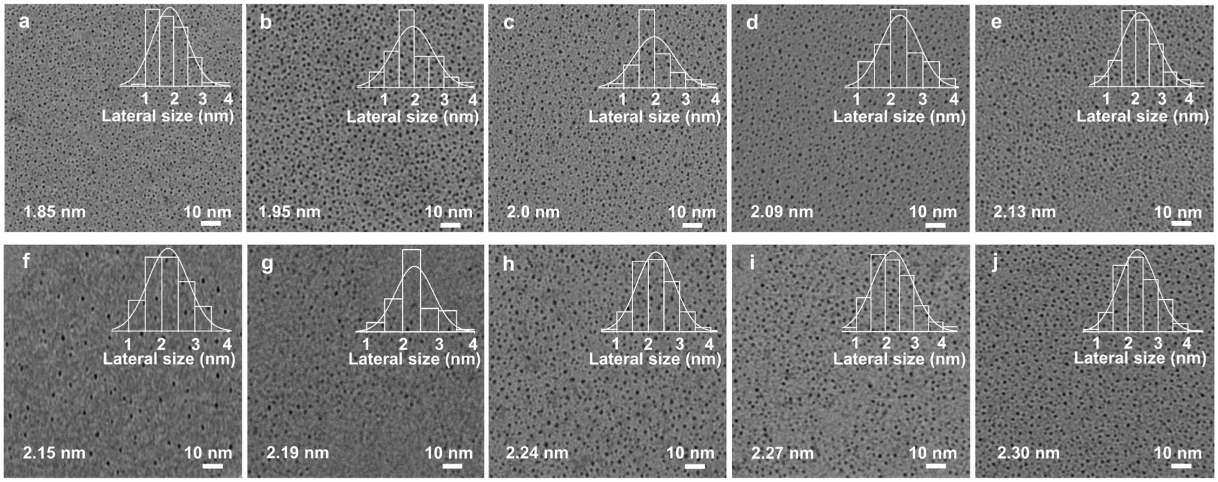

Supplementary Fig. 24 | TEM characterizations of full-color fluorescent CQDs. TEM images and corresponding lateral size distributions of p-CQDs (**a**), b-CQDs (**b**), c-CQDs (**c**), dc-CQDs (**d**), g-CQDs (**e**), yg-CQDs (**f**), y-CQDs (**g**), o-CQDs (**h**), or-CQDs (**i**) and r-CQDs (**j**), respectively. Numbers displayed in the bottom left corner of each image corresponds to the average lateral sizes of CQDs.

Supplementary Table 1 | Value ranges of input synthesis parameters. The value ranges of synthesis parameters are determined on the configuration of synthesis devices in the laboratories. Compared to our previous study of CQDs[1], we expand the list of candidate solutions and catalysts to cater for the enhanced complexity of the targeted problem involving multiple desired properties. Type of catalyst and type of solution are ranked by polarity, whereas lower polarity leads to smaller indexes. The precursor used in our experiments is 2,7-naphthalenediol molecule.

**Parameter**	**Min**	**Max**	**Increment**	**# of Possible Points**
Reaction temperature (ºC)	80	220	20	8
Reaction time (hr)	1	12	1	12
Ramp rate (ºC/min)	2	8	2	4
Mass of precursor (g)	0.02	0.2	0.02	10
Types of catalyst	EDA, H_2SO_4, HAc, urea			4
Volume/mass of catalyst (μL)/(mg)	0	1000	20	51
Types of solution	ethanol, deionized water, DMF, toluene, formamide			5
Volume (mL)	2	10	2	5
**Total # of Possible Points**				19,584,000

Supplementary Table 2 | Value ranges of colors defined by PL wavelength.

Target Properties		Value Range
PLQY		[0, 1]
Color	Purple	PL < 420 nm
	Blue	420 nm ≤ PL < 460 nm
	Cyan	460 nm ≤ PL < 490 nm
	Green	490 nm ≤ PL < 520 nm
	Yellow	520 nm ≤ PL < 550 nm
	Orange	550 nm ≤ PL< 610 nm
	Red	610 nm ≤ PL

Supplementary Table 3 | PL scan conditions and PLQY data for full-color fluorescent CQDs.

Samples	Excitation (nm)	Emission (nm)	PL range (nm)	PLQY (%)
p-CQDs	370	410	380-500	71
b-CQDs	355	420	365-550	51
c-CQDs	430	470	440-600	59
dc-CQDs	445	485	460-670	83
g-CQDs	375	490	400-700	58
yg-CQDs	480	530	490-700	65
y-CQDs	480	540	490-700	55
o-CQDs	530	575	540-750	46
or-CQDs	565	605	550-800	88
r-CQDs	605	645	608-750	74

Supplementary Table 4 | Recent progress of PL wavelengths of red CQDs.

Samples	PL wavelength (nm)	Ref.
r-CQDs	645	This work
r-CQDs	620	7
r-CQDs	622	8
r-CQDs	625	9
r-CQDs	635	10
r-CQDs	625	11
r-CQDs	620	12
r-CQDs	621	13
r-CQDs	639	14
r-CQDs	635	15

Supplementary Table 5 | The energy levels of full-color fluorescent CQDs.

Samples	HOMO (eV)	LUMO (eV)	λ_{edge} (nm)	$1240/\lambda_{edge}$ (eV)
p-CQDs	-6.52	-3.5	410	3.02
b-CQDs	-6.20	-3.25	420	2.95
c-CQDs	-6.72	-3.77	472	2.63
dc-CQDs	-6.92	-4.34	480	2.58
g-CQDs	-6.54	-4.01	490	2.53
yg-CQDs	-6.48	-4.10	520	2.38
y-CQDs	-6.34	-4.06	545	2.28
o-CQDs	-6.64	-4.63	600	2.01
or-CQDs	-6.60	-4.63	630	1.97
r-CQDs	-6.94	-5.03	650	1.91

Supplementary References

1. Han, Y. et al. Machine-learning-driven synthesis of carbon dots with enhanced quantum yields. *ACS Nano* **14**, 14761-14768 (2020).
2. Lu, S. et al. Accelerated discovery of stable lead-free hybrid organic-inorganic perovskites via machine learning. *Nat. Commun.* **9**, 3405 (2018).
3. Xue, D. et al. Accelerated search for materials with targeted properties by adaptive design. *Nat. Commun.* **7**, 11241 (2016).
4. Rumelhart, D. et al. Learning representations by back-propagating errors. *Nature* **323**, 533-536 (1986).
5. Yuan, R. et al. Accelerated discovery of large electrostrains in $BaTiO_3$-based piezoelectrics using active learning. *Adv. Mater.* **30**, 1702884 (2018).
6. Sun, S. et al. Accelerated development of perovskite-inspired materials via high-throughput synthesis and machine-learning diagnosis. *Joule* **3**, 1437-1451 (2019).
7. Zhang, Q. et al. Photoluminescence mechanism of carbon dots: triggering high-color-purity red fluorescence emission through edge amino protonation. *Nat. Commun.* **12**, 6856 (2021).
8. Medina-Lopez, D. et al. Interplay of structure and photophysics of individualized rod-shaped graphene quantum dots with up to 132 sp^2 carbon atoms. *Nat. Commun.* **14**, 4728 (2023).
9. Shi, Y. et al. Red phosphorescent carbon quantum dot organic framework-based electroluminescent light-emitting diodes exceeding 5% external quantum efficiency. *J. Am. Chem. Soc.* **143**, 18941-18951 (2021).
10. Wang, B. et al. Rational design of multi-color-emissive carbon dots in a single reaction system by hydrothermal. *Adv. Sci.* **8**, 2001453 (2020).
11. Zheng, Y. et al. Multicolor carbon dots prepared by single-factor control of graphitization and surface oxidation for high-quality white light-emitting diodes. *Adv. Optical Mater.* **9**, 2100688 (2021).
12. Madonia, A. et al. Dye-derived red-emitting carbon dots for lasing and solid-state lighting. *ACS Nano* **17**, 21274-21286 (2023).
13. Ding, H. et al. Large scale synthesis of full-color emissive carbon dots from a single carbon source by a solvent-free method. *Nano Res.* **15**, 3548-3555 (2021).
14. Chen, J. et al. Controlled synthesis of multicolor carbon dots assisted by machine learning. *Adv. Funct. Mater.* **33**, 2210095 (2023).

15. Sun, S. et al. Tumor microenvironment stimuli-responsive fluorescence imaging and synergistic cancer therapy by carbon-dot-Cu^{2+} nanoassemblies. *Angew. Chem. Int. Ed.* **59**, 21041-21048 (2020).
